# Core-shell 3D printed biodegradable calcium phosphate cement—Alginate scaffolds for possible bone regeneration applications

**DOI:** 10.3389/fddev.2024.1407304

**Published:** 2024-05-17

**Authors:** Clara Schweiker, Sergej Zankovic, Anna Baghnavi, Dirk Velten, Hagen Schmal, Ralf Thomann, Michael Seidenstuecker

**Affiliations:** ^1^ G.E.R.N. Tissue Replacement, Regeneration and Neogenesis, Department of Orthopedics and Trauma Surgery, Medical Center-Albert-Ludwigs-University of Freiburg, Faculty of Medicine, Albert-Ludwigs-University of Freiburg, Freiburg, Germany; ^2^ Institute for Applied Biomechanics, Faculty of Mechanical and Process Engineering, Offenburg University, Offenburg, Germany; ^3^ Department of Orthopedics and Trauma Surgery, Medical Center-Albert-Ludwigs-University of Freiburg, Faculty of Medicine, Albert-Ludwigs-University of Freiburg, Freiburg, Germany; ^4^ Freiburg Center for Interactive Materials and Bioinspired Technologies (FIT), Albert-Ludwigs-University Freiburg, Freiburg, Germany

**Keywords:** 3D printing, CPC, core-shell printing, alginate, self-setting, scaffold, bone regeneration

## Abstract

The core/shell 3D printing process using CPC and alginate is intended to create biodegradable scaffolds that have a similar stability to bone tissue and also offer sufficient and continuous antibiotic release. In this way, a patient-specific and patient-friendly process will be established, which should optimally support the human organism in its regeneration. To generate the best possible strength values, the printed scaffolds underwent various post-treatments and were then tested in a material test. The test methods included self-setting, storage in a drying cabinet with a water-saturated atmosphere at 37°C, followed by incubation in PBS, freeze-drying, and coating the samples with alginate. Additionally, a degradation test at pH 7.4 and pH 5 was carried out to test stability under *in vitro* conditions. It was shown that the untreated and freeze-dried samples failed at a maximum load of 30–700 N, while the remaining scaffolds could withstand a load of at least 2,000 N. At this failure load, most of the test series showed an average deformation of 43.95%. All samples, therefore, remained below the strength of cancellous bone. However, based on a 20% load after surgery, the coated scaffolds represented the best possible alternative, with a Young’s modulus of around 1.71 MPa. We were able to demonstrate that self-setting occurs in core-shell printed CPC/alginate scaffolds after only 1 day, and that mass production is possible. By coating with alginate, the compressive strength could be increased without the need for additional post-treatment. The mechanical strength was sufficient to be available as a scaffold for bone regeneration and additionally as a drug delivery device for future applications and experiments.

## 1 Introduction

Fractures that result in significant bone defects or even bone loss disrupt the supply of blood and nutrients to the injured area, as the surrounding blood vessels are usually also affected. Medication administered orally or intravenously cannot reach the site in sufficient concentration to ensure an optimal course of treatment (Kanellakopoulou and Giamarellos-Bourboulis, 2000; Kluin et al., 2013). With local application of antibiotics, concentrations up to 25 to 250 times higher can be achieved in the immediately surrounding tissue without exerting harmful effects on the rest of the organism (Kanellakopoulou and Giamarellos-Bourboulis, 2000; Kluin et al., 2013; Seidenstuecker et al., 2017; Rawson et al., 2021). So far, several products have attempted to tackle this problem. One such current system is based on collagen. However, collagen is not a suitable bone replacement material (Kanellakopoulou and Giamarellos-Bourboulis, 2000). It differs too much from bone in its Young’s modulus. Das et al. (2024) described a 3D printed nanocomposite hydrogel based on borophene for controlled release.

Another more bone-like system is based on synthetic polymers with a lactic or glycolic acid base. However, these systems produce acidic degradation products (Alexis, 2005; Liu et al., 2006), which reduce the pH value in the surrounding tissue and thus promote inflammatory reactions and the further degradation of bone material (Kluin et al., 2013).

Above these products are Calcium phosphate cements (CPC) used in bone regeneration. They combine bone-like properties with non-toxic degradation products and is therefore very suitable as a bone replacement material. According to van de Belt et al. (2000), around 90% of orthopaedic surgeons in the United States use antibiotic-loaded bone cement. However, these systems also have their downsides. This, and all other systems listed so far, have poor release kinematics for the incorporated antibiotics (Kanellakopoulou and Giamarellos-Bourboulis, 2000; van de Belt et al., 2000; Díez-Peña et al., 2002). The minimum inhibitory concentration is often not reached (van de Belt et al., 2000). These sub-inhibitory concentrations can lead to the promotion of antibiotic resistance (van de Belt et al., 2000). Additionally, the antibiotic is mixed directly into the cement matrix, which can reduce strength by up to 30% (Vorndran et al., 2013). Furthermore, none of these systems is used as a pre-printed scaffold, which would allow patient-specific implants.

Another advantage of CPC-scaffolds is the osteoinductive effect if the scaffold is printed within a critical size diameter of 10 mm (Meininger et al., 2019).

To achieve this, a core/shell 3D-printing (Raja et al., 2021; Kilian et al., 2022) process is being established, in which alginate forms the core layer as an antibiotic carrier system and is encased in CPC, using a co-axial print head. This printing process offers not only the possibility of producing patient-specific implants (Guzzi and Tibbitt, 2020), e.g., from CT/MRI data, but also of implanting individually composed pharmaceuticals. This would support the global trend towards individualized therapy. Accordingly, a personalized and patient-friendly procedure is being established, which should support the human organism in its regeneration in the best possible way.

In particular, 3D printing of calcium phosphate cement, which sets to calcium-deficient hydroxyapatite (CDHA) (Vorndran et al., 2013; Lin et al., 2021), is very suitable for bone regeneration, because the bone is also made of HA. In a previous work we could already prove that just the choice of geometry (rotationally symmetric) can lead to an increased mechanical strength (Bertrand et al., 2023), even higher than comparable sintered ceramics. In this work, the core-shell printing of CPC with alginate gel in the core is considered, with the background that alginate requires free Ca^2+^ ions for crosslinking (with release of water) and CPC requires water for setting. Particular attention will be paid to the formation of surface cracks which leads to less mechanical stability, as our working hypothesis is that crosslinking/setting occurs from the inside out. A mutual cross-linking/setting reaction alginate/CPC by the Ca^2+^ originating from the CPC for the cross-linking of the alginate and the water originating from the cross-linking for the setting of the CPC has not yet been described.

## 2 Materials and methods

The printer needle with 0.3 mm (article No. 500883) inner diameter was purchased from VIEWEG GmbH (Kranzberg, Germany) and the needle with an inner diameter of 1.36 mm (article No. 7018068) was purchased from Nordson EFD (Ohio, United States). Sodium alginate article No. (BCCB8704) was purchased by Sigma-Aldrich (now Merck, Darmstadt, Germany). The CPC paste for printing (20 mL, article No. 087-020-PL) was purchased from Innotere (Radebeul Germany). For post-treatment the Phosphate Buffered Saline (PBS) (article No. RNBL9592) was purchased from Thermo Fischer Scientific (Darmstadt, Germany) and the TRIS-hydrochlorid Pufferan (≥99%, article No. 9090.3) as well as the sodium hydroxide (article No. K021.1) were both purchased from Carl Roth (Karlsruhe, Germany).

### 2.1 3D printing

**FIGURE 1:**
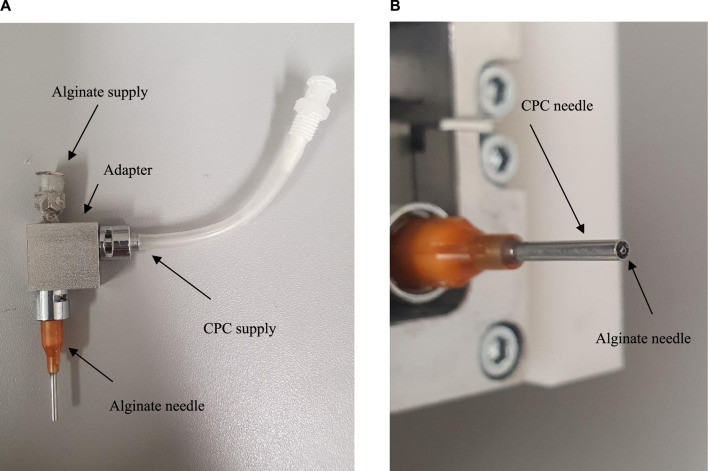
Modified Core-shell printing head for printing CPC; **(A)** Modification for printing CPC; **(B)** Core/Shell Needles.

3D printing was performed on a 3D Bioplotter (Envisiontec GmbH, Gladbeck, Germany). The core-shell print head was modified by us so that it can also print CPC using a outer needle (Vieweg) and alginate by using a full dispensing needle (Vieweg), which was mounted within the center of the conical needle (Figure 1). A 0.3 mm inner diameter (ID) needle for the 3% w/v alginate solution and a 1.36 mm ID needle for the CPC plotter paste to print the scaffold geometry were used.

### 2.2 Printing parameters

As we have seen in previous works (Blankenburg et al., 2022; Bertrand et al., 2023) that the parameters always depend on the geometry, the optimization was done directly on the geometries to be printed, instead of using the “Parameter Tuning” function, which prints only lines, as the printer software provides. The optimization of the parameters was carried out for both the shell: i.e., the CPC and the inner core: alginate, so that no printing errors occurred with either. Therefore, the scaffolds could be printed with much smaller needle inner diameters. The parameters for the new printing process must be set so that the high viscosity of the CPC does not prevent the printing of the low-viscosity alginate. In reverse, the alginate should not break through the outer CPC shell. To achieve this the following printing parameters were varied:

• Pressure Alginate [bar]: 0.5–1.2
• Pressure CPC [bar]: 1.5–3.5
• Printing speed [mm/s]: 5–9
• Pre-flow Alginate [s]: 0.11–1.4
• Pre-flow CPC [s]: 0.1–1.55
• Post-flow Alginate [s]: −0.55–0
• Post-flow CPC [s]: −1.20–0.05

The needle offset [mm] does not need to be varied, because it is calculated from 80% of the needle inner diameter. Unlike in previous works (Blankenburg, Vinke et al., 2022; Huber et al., 2022), it was not possible to identify the printing parameters using the “Parameter Tuning” of the “Visuals Machines” software. This program does not take into account the required difference in pressure of the different materials, which is why they were tested directly in a modeled geometry.

### 2.3 Geometry

**FIGURE 2:**
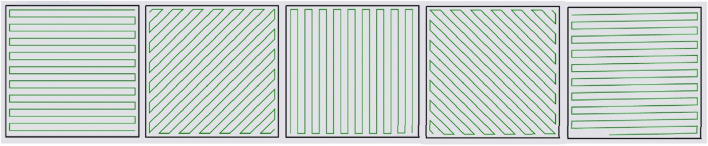
Rotation of the inner structure.

Creo Parametric 10.0.0.0 (PTC Inc., Las Vegas, United States), was used to create the geometry. For this purpose, a disk with a diameter of 15 mm was created. The model is extruded to 1 mm according to the needle offset. In addition, an internal structure was designed in “Visual Machines” (Envisiontec GmbH, Gladbeck, Germany), which should resemble the trabecular structure of the cancellous bone. In this case, a serpentine structure was selected (Figure 2), as this reduced printing errors. To ensure that the trabecular structure was also pronounced in different directions in 3D models, the model including the inner structure had to be offset by a certain angle for each layer.

### 2.4 Post-treatment

A total of twelve groups with at least five samples each were prepared according the DIN ISO for mechanical testings. Table 1 shows all groups with their respective post-treatment. The aim is to investigate how the different post-treatments affect the strength. The incubation takes place in a chamber with double-distilled water at 37°C, which forms a water-saturated atmosphere. The incubation in PBS however takes places at room temperature. The freeze-dried samples were exposed to a vacuum of 0.140 mbar and a temperature of −80°C. Samples, which were placed in liquid nitrogen for about 40 s were broken immediately afterwards for sample characterization. Each sample of the alginate-coated group, were covered with a different alginate-solution varying in a range of 1% w/v to 3% w/v.

**TABLE 1:** Classification of the groups according to post treatment.

Group post treatment	GP1	GP2	GP3	GP4	GP5	GP6	GP7	GP8
(Self)Setting/crosslinking for 1d	X							X
Water-saturated atmosphere 3d		X		X	X	X	X	
PBS 1 week		X			X	X		
TRIS pH5 2 weeks						X	X	
TRIS pH 7.4 2 weeks				X	X			
Freeze			X					
Alginate coating								X

### 2.5 Degradation test

In addition to the PBS post-treatment, a degradation test was performed according to ISO EN 10993. The required TRIS buffer was also prepared according to the specifications. We supplemented this test because ISO EN 10993 only specifies a pH of 7.4. To simulate the conditions in the human body caused by inflammation, the experiment was repeated with TRIS buffer pH 5.0. However, the incubation in TRIS was initially performed for only 14 days.

### 2.6 Sample characterization

The dimensions of the scaffolds were measured after the post-treatment with a digital caliper gauge (Precise PS 7215, Burgwächter, Wetter-Volmarstein, Germany). After the respective post-treatment, the samples were characterized by 3D Laser scanning microscopy (Keyence VK-X 200; Keyence, Osaka, Japan) at ×200 magnification according their strand thickness. This was to find out whether the samples swell or shrink as a result of their post-treatment. All samples were compared with the untreated reference sample.

The samples were also examined under the scanning electron microscope ESEM (FEI Quanta 250 FEG, FEI, Hilsboro; OR, United States) with an EDX unit (Oxford Instruments, Abingdon, United Kingdom). To analyze the surface properties a Large Field Detector (LFD) and a Backscattered Electron Detector (vCD) were used. The aim was to investigate whether the surface cracks from previous works could be minimized through the use of alginate. In addition, the fracture point and thus the alginate layer on the inside was examined to determine whether it was printed evenly and in the correct place.

The microscope examination was followed by material testing. The finished printed and post-treated geometries were loaded with a pre-load of 1 N on the Zwick Z005 universal testing machine (Zwick/Roell, Ulm, Germany) and then tested at a speed of 1 mm/s until failure. The maximum test load was set to 2,000 N, the maximum deformation to 50%.

### 2.7 Statistics

In accordance with DIN standards (DIN EN 843-2; DIN EN ISO 527; DIN EN ISO 6892-1) for mechanical testing, at least five samples were mechanically tested. All results are expressed as means ± standard deviations. Measured values were analyzed using ANOVA (Fisher-LSD) with a significance level of *p* < 0.05. Origin 2023 Professional SR1 (OriginLab, Northampton, MA, United States) was used for all statistical analyses.

## 3 Results

### 3.1 Scaffold dimensions

The following results were obtained for the dimensions (external and internal) of the scaffolds after 3D printing and post-processing. The scaffolds had a diameter of 15.6 ± 0.26 mm and a height of 4.51 ± 0.31 mm. The sample with the smallest diameter measured 15.2 mm, while the largest was 16.1 mm in diameter. The lowest height recorded was 4.11 mm, and the highest was 4.64 mm. The variation in diameter and height was due to printing errors, such as material remaining in the last position.

**FIGURE 3:**
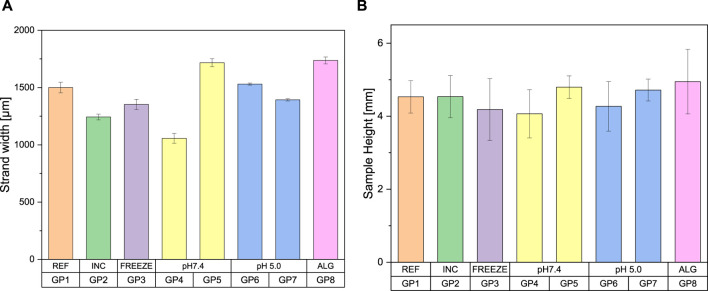
Bar charts as a function of the post-treatment of the samples for **(A)** strand width **(B)** sample height, classification of the groups according to Table 1 (*n* = 5)

All values range from 1,057 to 1,737 µm (Figure 3). The sample coated with a 3% alginate solution showed a strand width approximately 15% thicker than the reference sample. Group 4 exhibited a significantly smaller strand width, which was also visible as material erosion on the water surface. This phenomenon was not observed for the other samples during the degradation tests, which showed correspondingly higher values for their strand width. The incubated and freeze-dried samples each show slightly thinner strand widths compared to the reference sample with 1,500 µm.

**FIGURE 4:**
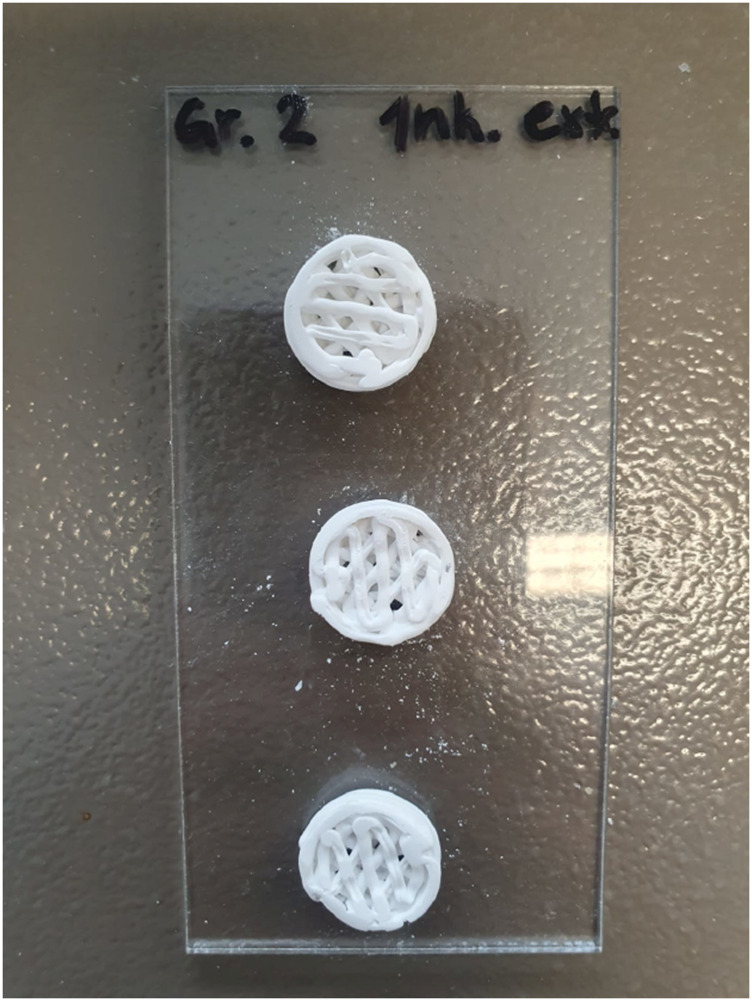
Exemplary illustration of the 3D-printed and post-processed scaffolds.

If the general sample heights were compared with each other, it becomes apparent that they vary with a similar fluctuation. They range from a height of 4.07–4.95 mm. The pictorial illustration is shown in Figure 3. Here too, group 4 is the sample with the greatest material removal and the coated sample with the greatest average height. The incubated sample with a value of 4.54 mm shows no significant difference to the reference sample with a height of 4.53 mm. The freeze-dried sample and the sample treated in pH 5 without PBS also show slight shrinkage compared to the reference sample. An exemplary illustration of some post-processed samples is shown in Figure 4.

### 3.2 Surface conditions

**FIGURE 5:**
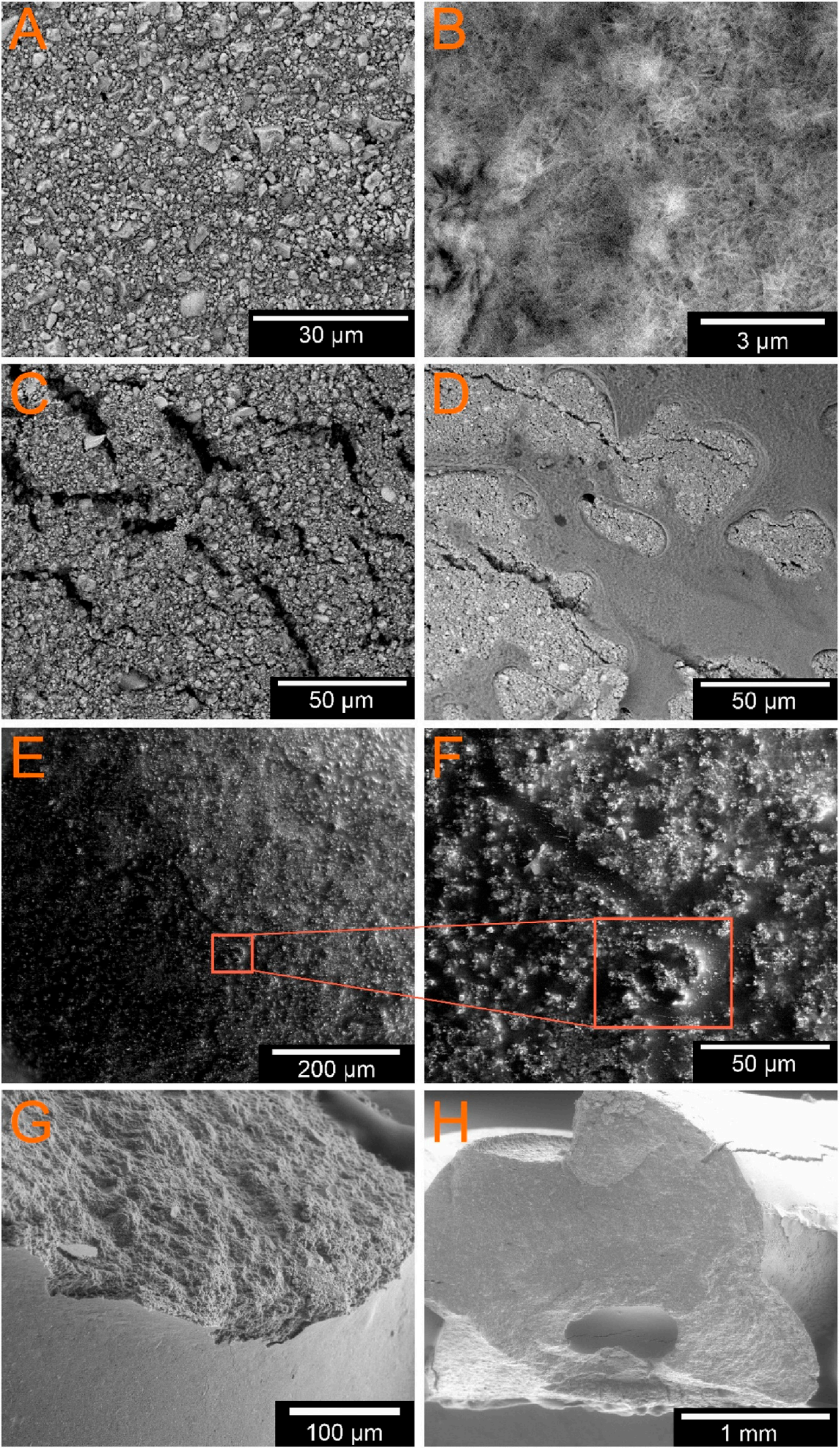
ESEM images of the different GP; **(A)** GP1 untreated sample; **(B)** GP2 in PBS incubated, and slightly visible HA layer; external alginate coating of **(C)** 1% w/v; **(D)** 3% w/v; **(E, F)** untreated freezed sample in different magnifications (×233 and ×930); **(G, H)** fractured samples directly after printing **(G)** and after lingering in water-saturated atmosphere for 3d **(H)**; the empty core can be clearly seen in **(G, H)**.

Using 3D laser scanning microscopy and scanning electron microscopy, the following surface properties of the scaffolds were determined. The untreated sample from group 1 shows a very homogeneous appearance (Figure 5A). It is relatively densely packed with few alginate lakes on the surface. The light-colored particles represent the calcium crystals. The incubated group is the only one to show a needle-like structure, which can be identified as a hydroxyapatite (HA) layer (Figure 5B). The images of the samples coated with alginate show different results depending on the solution concentration. GP8-1 was coated with a 1% w/v alginate solution. It has a very rough surface with many cracks (Figure 5C). However, the images showed no signs of alginate coating. The samples GP8-2 and GP8-3 were coated with a 2% w/v and 3% w/v alginate solution. Both show a very smooth surface in places, which is indicative of the alginate layer. However, the samples were largely covered by alginate, but relatively unevenly (Figure 5D). Some areas have thicker layers of alginate than others. The coating layer itself also shows a somewhat granular structure. Some cracks are also visible under the alginate layer, into which the alginate penetrates or partially covers. The freeze-dried sample from group 3 shows superficial cracks. Figures 5E, F shows that the crack was filled with alginate and only individual calcium particles were washed along with it.

The SEM images in Figures 5G, H do not show any significant material contrast between the inner and outer layers in both samples. In both samples examined, a hole can be seen in a single strand. The sample were examined without the vCD attachment in order to obtain a larger image section and to be able to recognize the strands more clearly. Therefore, the difference in dimensions cannot be shown as clearly in this image. Nevertheless, a cavity filled with alginate is clearly recognizable with a size of 368.4 µm (Figure 5H).

In addition, the empty core can be clearly seen in Figures 5G, H. Due to the necessary drying process before taking the SEM images, the water evaporated. The alginate is not visible due to the low mass fraction.

#### 3.2.1 Mechanical properties

**FIGURE 6:**
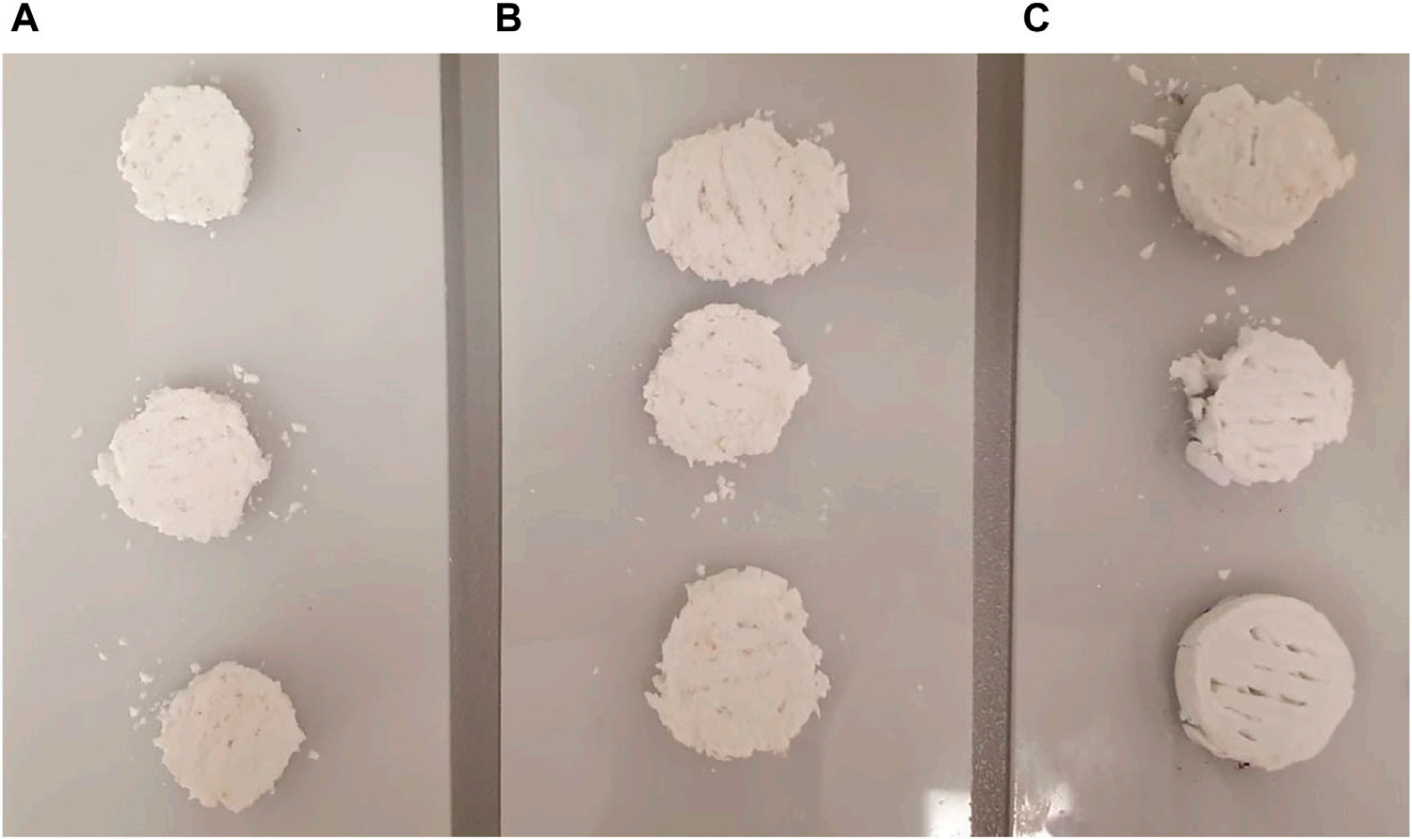
Selection of different samples after mechanical testing; **(A)** GP5; **(B)** GP6; **(C)** GP8.

Tensile/compression tests were performed on the universal testing machine to determine the mechanical properties of the different scaffolds. It can be observed that the samples in GP1 (reference) and GP3 (freeze-dried) exhibit significantly lower maximum values. Compared to the other test series, these were significantly weaker. This is evidenced by the fact that the samples were only compressed but not broken. In all post-treated samples, it can be seen that only the outer ring breaks during material failure. Despite the fact that samples of GP6 have not undergone any further post-treatment apart from the (self)setting/crosslinking and alginate coating, they exhibit significantly less deformation with only 15% at the same maximum load of around 2,000 N. Other groups show a deformation between 30% and 50%. This difference is evident in their subsequent height. The samples of GP8 only show a few indentations, but not concrete fracture points at the edge, unlike the other groups (see Figure 6). The individual strength values as well as the calculated compressive strength and Young’s modulus were listed again in Table 2 below.

**TABLE 2:** Comparison of the mechanical properties of the different groups (*n* = 5).

	Maximum failure load [N]	Compressive strength [MPa]	Young´s modulus [MPa]
GP1	412.6 ± 172.4	2.3 ± 1.0	0.3 ± 0.2
GP2	2081.6 ± 167.9	11.78 ± 1.0	1.0 ± 0.4
GP3	90.1 ± 7.51	0.5 ± 0.04	0.04 ± 0.02
GP4	2408.5 ± 536.8	13.6 ± 3.0	0.6 ± 0.2
GP5	2,189.7 ± 67.9	12.4 ± 0.4	1.2 ± 0.1
GP6	2,149.4 ± 44.4	12.2 ± 0.3	1.1 ± 0.2
GP7	2,109.1 ± 29.2	11.9 ± 0.2	0.9 ± 0.2
GP8	2031.8 ± 21,0	11.5 ± 0.1	1.3 ± 0.5

The non-post-treated sample GP1 showed a 4-fold higher mechanical strength compared to the GP3 freeze-dried sample, which also had no (self) setting/crosslinking time. The post-incubated samples in PBS but also TRIS showed up to 20 times higher compressive strengths. However, no significant differences has been found between incubation in PBS and TRIS (regardless of the pH value). In contrast, the external alginate coatings do not result in any increase in mechanical strength compared to the samples incubated in the water-saturated atmosphere or additionally post-solidified in PBS. This means that the external alginate coating alone, without further post-treatment, results in the same compressive strength of the core/shell printed samples (with significant time savings).

## 4 Discussion

### 4.1 Geometry

No definitive statement can be made about the influence of PBS on the sample diameter, strand thickness or sample height. In a direct comparison, the samples treated with PBS showed a higher average profile of around 10%. However, a comparison of the diameter, strand widths of the samples treated with and without PBS showed no clear difference. Based on the results, it can be assumed that although post-treatment with three-day incubation or freeze-drying leads to shrinkage of the strands, it has no negative influence on the height profile of the scaffolds. However, the samples from the degradation tests only showed an average decrease of around 5% in direct comparison. This indicates that mass erosion takes place during the test (Burkersroda et al., 2002; Blankenburg et al., 2022). Iglesias-Mejuto and García-González (2021) investigated the influence of HA, alginate and Ca^2+^ concentrations on alginate aerogel scaffolds. It was shown that the addition of HA leads to less volume shrinkage.

### 4.2 Surface condition

The results from the SEM have shown that the HA layer is the only one to form in the incubated samples from groups 2. This may be due to the one-week incubation in PBS (Seidenstuecker et al., 2018). The samples of the groups coated with different alginate solutions show different results according to their solution. Samples from group 8-1 show no signs of alginate coating in the SEM, as the solution is too thin to be detected in the ESEM. Group 8-2 and 8-3, on the other hand, both show an alginate coating, albeit unevenly. However, all groups show some surface cracks. As all scaffolds have also undergone a curing reaction, it can be concluded that there must be an alginate layer inside that triggers this reaction. Previous tests have shown that air humidity at room temperature is not sufficient for prompt setting. However, the fractured samples do not suggest that the core layer was printed continuously and evenly. Only one of the strands has a cavity, which is filled with alginate. The cavity with a diameter of 368.4 µm matches the inner needle diameter of 0.3 mm used. Additionally, it can be seen that the alginate core is not centered in the CPC sheath, which leads to an uneven wall thickness of the individual strands. Unlike the research group of Mirek et al. (2022), we did not wait for each layer to solidify before printing the next one. As can be seen in the microscope images, the individual strands have therefore fused together in this study, which may have an effect on the inner alginate layer.

### 4.3 Mechanical properties

The reason for the low strength of sample GP3 is that this sample was frozen directly after printing to prevent the (self) setting/crosslinking reaction and to be able to compare it with the other samples. A direct comparison with GP1 confirms our initial hypothesis—a self-induced setting reaction took place within 1 day. However, a comparison with the post-treated samples shows that this setting reaction appears to be far from complete.

You can also see the deformation of the strand (Figures 5G, H) during the printing process (from round to oval) and the overlay of 2 layers, which resulted in one of two core structures (see the existing cavity) being overprinted by the overlay. This ultimately meant that only one of the two core structures survived. For future printing processes, the overlay should be reduced from 20% to 15%.

If all the values determined for the Young’s modulus are compared with the mechanical properties of the cancellous bone, it can be seen that all samples have strength values that are 10–5,000 times lower than those of human bone (Epple, 2003). Due to the fact that the scaffolds are to be used for small bone defects, the full load cannot be assumed here. For example, patients are not allowed to put full weight on the affected side for 6 weeks after an implantation. A maximum load of 20% is often recommended. This would correspond to a compressive strength of only 2–1,000 MPa. The highest possible compressive strength among the samples is currently 12.2 MPa, which is in the lower range of bone strength and is therefore suitable for use in the body. Material testing has shown that only the outer edge of most scaffolds breaks under pressure. The inner structure, which is intended to imitate the trabecular structure of cancellous bone, remains intact. The behavior of the alginate-coated specimens is somewhat different. Although these do not exhibit the highest maximum failure loads, it has been shown that they are subject to significantly less deformation at a comparable maximum load. Despite the fact that the examination of the surface showed more cracks than the untreated sample, for example, it has the best strength values in terms of deformation. Coating with a 3-% w/v alginate solution therefore results in less deformation of the sample under the same maximum load than comparable samples, but with great time savings, as post-treatments such as storage in a water-saturated atmosphere (3 days) and/or post-solidification in PBS (1 week) become unnecessary.

If we now compare the strength values determined with those from previous work (Bertrand et al., 2023) in this research group, it becomes clear that there is also a significant difference here. The maximum failure load is within a similar range. However, the calculated compressive strength and the Young’s modulus are considerably lower. The reason for this is the difference in the cross-sectional area. The samples from previous tests had a cross-section of 10.5 mm, whereas the scaffolds in this test series have a diameter of 15 mm (Bertrand et al., 2023).

Additionally, Bertrand et al. (2023) used a different internal structure. This shape resembles a helical structure in its extruded form and is therefore much more stable than superimposed lines at a 45° angle in the present work. Similarly, no alginate was used in a core-shell printing process in the previous experiments. This new printing process now results in strand widths that are about 2–8 times larger than before. In addition, four times fewer layers are required to achieve the same sample height. This results in a less stable internal structure.

In the degradation test, no influence of the TRIS buffer (regardless of whether pH 7.4 or pH 5) on the mechanical properties of the scaffolds could be determined. In contrast to a previous study (Seidenstuecker et al., 2021) in which incubation in the TRIS buffer affected the strength of the scaffolds and reduced it by a third from ∼1000 N to ∼650 N. However, in contrast to the present work, the previous work involved beta tricalcium phosphate, which has a higher degradation rate than HA (Epple, 2003).

The addition of an alginate core layer ensures automatic initiation of the setting reaction, but does not necessarily guarantee greater stability (Vorndran et al., 2013). Especially since it was found that the alginate layer is not continuous. On the one hand, it is possible that the core layer was not printed continuously; or the two materials can also mix with each other in places. The mixing of the alginate with the cement matrix can therefore also lead to a reduction in strength. This phenomenon has already been observed by the Vorndran et al. (2013) working group. However, the addition of alginate can lead to an increase in strength (Lee et al., 2011). The values of compressive strength in our study were around 10 MPa. They are comparable to the calculated results of our work with an average of 12.23 MPa. When looking at the compressive strength of alginate alone, as Zhang et al. (2019) values of only 1.5–14.2 kPa, depending on the composition, were found here. In combination with CPC, the value in our and Lee et al.’s (2011) approach was 1,000 times higher and was in the range of bone.

## 5 Conclusion

We were able to show that self-setting occurs in core-shell printed CPC/alginate scaffolds after only 1 day and that mass production is possible. By coating with alginate, the compressive strength could be increased without the need for additional post-treatment. The mechanical strength was sufficient to be available a scaffold for bone regeneration (and additionally drug delivery device for future applications and experiments). As an outlook for future research, the next step would be to add active substances such as antibiotics or growth factors to the alginate and carry out release tests to determine the quantities released by means of HPLC.

## Data Availability

The raw data supporting the conclusion of this article will be made available by the authors, without undue reservation.
