# Corrigendum: Core-shell 3D printed biodegradable calcium phosphate cement – Alginate scaffolds for possible bone regeneration applications

**DOI:** 10.3389/fddev.2024.1452132

**Published:** 2024-07-18

**Authors:** Clara Schweiker, Sergej Zankovic, Anna Baghnavi, Dirk Velten, Hagen Schmal, Ralf Thomann, Michael Seidenstuecker

**Affiliations:** ^1^ G.E.R.N. Center of Tissue Replacement, Regeneration and Neogenesis, Department of Orthopedics and Trauma Surgery, Medical Center-Albert-Ludwigs-University of Freiburg, Faculty of Medicine, Albert-Ludwigs-University of Freiburg, Freiburg, Germany; ^2^ Institute for Applied Biomechanics, Faculty of Mechanical and Process Engineering, Offenburg University, Offenburg, Germany; ^3^ Department of Orthopedics and Trauma Surgery, Medical Center-Albert-Ludwigs-University of Freiburg, Faculty of Medicine, Albert-Ludwigs-University of Freiburg, Freiburg, Germany; ^4^ Freiburg Center for Interactive Materials and Bioinspired Technologies (FIT), Albert-Ludwigs-University Freiburg, Freiburg, Germany

**Keywords:** 3D printing, CPC, core-shell printing, alginate, self-setting, scaffold, bone regeneration

In the published article, there was an error in Table 1 as published. An X was incorrectly placed at GP4 for PBS 1 week. The corrected Table 1 and its caption Table 1: Classification of the groups according to post treatment appear below.

**TABLE 1:** Classification of the groups according to post treatment.

Group post treatment	GP1	GP2	GP3	GP4	GP5	GP6	GP7	GP8
(Self)Setting/crosslinking for 1d	X							X
Water-saturated atmosphere 3d		X		X	X	X	X	
PBS 1 week		X			X	X		
TRIS pH5 2 weeks						X	X	
TRIS pH 7.4 2 weeks				X	X			
Freeze			X					
Alginate coating								X

In the published article, there was an error. In several instances, GP4 was incorrectly used instead of GP3.

A correction has been made to **Results**, *3.2.1 Mechanical properties*, Paragraph 1. This sentence previously stated:

“It can be observed that the samples in GP1 (reference) and GP4 (freeze-dried) exhibit significantly lower maximum values.”

The corrected sentence appears below:

“It can be observed that the samples in GP1 (reference) and GP3 (freeze-dried) exhibit significantly lower maximum values.”

A correction has been made to **Results**, *3.2.1 Mechanical properties*, Paragraph 2. This sentence previously stated:

“The non-post-treated sample GP1 showed a 4-fold higher mechanical strength compared to the GP4 freeze-dried sample, which also had no (self) setting/crosslinking time.”

The corrected sentence appears below:

“The non-post-treated sample GP1 showed a 4-fold higher mechanical strength compared to the GP3 freeze-dried sample, which also had no (self) setting/crosslinking time”

A correction has been made to **Discusson**, *4.3 Mechanical properties*, Paragraph 1. This sentence previously stated:

“The reason for the low strength of sample GP4 is that this sample was frozen directly after printing to prevent the (self) setting/crosslinking reaction and to be able to compare it with the other samples.”

The corrected sentence appears below:

“The reason for the low strength of sample GP3 is that this sample was frozen directly after printing to prevent the (self) setting/crosslinking reaction and to be able to compare it with the other samples.”

In the published article, there was an error. Group 12 was incorrectly used instead of Group 8.

A correction has been made to **Discusson**, *4.2 Surface condition*. This sentence previously stated:

“Samples from group 12-1 show no signs of alginate coating in the SEM, as the solution is too thin to be detected in the ESEM. Group 12-2 and 12-3, on the other hand, both show an alginate coating, albeit unevenly.”

The corrected sentence appears below:

“Samples from group 8-1 show no signs of alginate coating in the SEM, as the solution is too thin to be detected in the ESEM. Group 8-2 and 8-3, on the other hand, both show an alginate coating, albeit unevenly.”

The authors apologize for these errors and state that they do not change the scientific conclusions of the article in any way. The original article has been updated.

